# SOCIAL NETWORK STRENGTHS: AN EXPLORATORY ANALYSIS OF A MEASURE OF TIES AMONG OLDER PEOPLE

**DOI:** 10.1093/geroni/igz038.640

**Published:** 2019-11-08

**Authors:** Raeann G LeBlanc, Lisa Chiodo, Cynthia Jacelon

**Affiliations:** University of Massachusetts Amherst, Amherst, Massachusetts, United States

## Abstract

Purpose: To explore a self-report measure of social network features among a sample of older people living with multiple chronic conditions based on the conceptual model of Social Network Influences on Health. Design: A cross-sectional descriptive study design using a telephone survey methodology was used. Methods: An exploratory principle component analysis with a varimax rotation was performed on items that measured the identified components (reciprocity, size, proximity, density, general activation, activation when sick, duration, closeness, involvement in health, interaction frequency) of social networks. All items were standardized prior to analyses. Results: Self-report social network data were collected from eighty-four older people living in the community and managing multiple chronic conditions. The principal components model, operationalized as tie strength, contained six items based on factorability: reciprocity, social network size, proximity, density and perception of the activation in general and when sick of close social network members. Results yielded acceptable factorability (KMO = 0.781, Bartlett p 0.70). Two components that had eigenvalues greater than 1.0, explained 61.7% of the total variance. The first factor was interpreted as total social network resources, while the second factor was identified as social network availability. Conclusion: Exploratory principal component analysis supports a measure of social network features, tie strength, that can be tested in future studies. Assessing these variables is useful in identifying specific relationship features critical to managing chronic conditions in older age and advances current measurement of social networks important to living well in older age.

